# LINC01355 suppresses breast cancer growth through FOXO3-mediated transcriptional repression of CCND1

**DOI:** 10.1038/s41419-019-1741-8

**Published:** 2019-06-26

**Authors:** Bolun Ai, Xiangyi Kong, Xiangyu Wang, Kai Zhang, Xue Yang, Jie Zhai, Ran Gao, Yihang Qi, Jing Wang, Zhongzhao Wang, Yi Fang

**Affiliations:** 1 Department of Breast Surgical Oncology, National Cancer Center/National Clinical Research Center for Cancer/Cancer Hospital, Chinese Academy of Medical Sciences and Peking Union Medical College, Beijing, China; 2 Department of Cancer Prevention, National Cancer Center/National Clinical Research Center for Cancer/Cancer Hospital, Chinese Academy of Medical Sciences and Peking Union Medical College, Beijing, China

**Keywords:** Cancer epigenetics, Long non-coding RNAs

## Abstract

Previously, several protein-coding tumor suppressors localized at 1p36 have been reported. In the present work, we focus on functional long non-coding RNAs (lncRNAs) embedded in this locus. Small interfering RNA was used to identify lncRNA candidates with growth-suppressive activities in breast cancer. The mechanism involved was also explored. LINC01355 were downregulated in breast cancer cells relative to non-malignant breast epithelial cells. Overexpression of LINC01355 significantly inhibited proliferation, colony formation, and tumorigenesis of breast cancer cells. LINC01355 arrested breast cancer cells at the G0/G1 phase by repressing CCND1. Moreover, LINC01355 interacted with and stabilized FOXO3 protein, leading to transcriptional repression of CCND1. Importantly, LINC01355-mediated suppression of breast cancer growth was reversed by knockdown of FOXO3 or overexpression of CCND1. Clinically, LINC01355 was downregulated in breast cancer specimens and correlated with more aggressive features. There was a negative correlation between LINC01355 and CCND1 expression in breast cancer samples. LINC01355 acts as a tumor suppressor in breast cancer, which is ascribed to enhancement of FOXO3-mediated transcriptional repression of CCND1. Re-expression of LINC01355 may provide a potential therapeutic strategy to block breast cancer growth and progression.

## Introduction

Breast cancer is the most common malignancy in females^1^. Despite noticeable advancement in detection and treatment, the disease is still devastating^2,3^. Understanding the molecular basis for the aggressive phenotype of breast cancer is critical to identify novel targets to improve therapeutic outcomes.

Abnormality of the chromosome 1p36 locus has been detected in many human cancers^4–6^. Nagai et al.^7^ reported a common deletion in the 1p36 region in breast cancer patients (10/44; 23%), suggesting that the region contains potential antitumor genes. Previously, a number of 1p36 genes have been identified as tumor suppressors, such as CAMTA1, AJAP1, and CASZ1^8–10^. Apart from the protein-coding genes, tumor suppressive non-coding genes are also presented in the 1p36 locus^6^. For instance, microRNA (miR)-34a, which is located in 1p36.23, has a pro-apoptotic role in neuroblastoma cells^11^.

Long non-coding RNAs (lncRNAs) are a large class of transcripts of longer than 200 bp and regulate a variety of biological processes, including cell proliferation, motility, and invasion^12^. Accumulating evidence has indicated that lncRNAs are involved in tumor development and progression^13^. For instance, lncRNA SNHG14 was found to confer trastuzumab resistance to breast cancer cells^14^. lncRNA MIR100HG shows the ability to promote breast cancer cell proliferation^15^. lncRNAs can regulate gene expression through multiple mechanisms. It has been documented that lncRNA can sponge specific miRs and indirectly modulate gene expression at the post-transcriptional level^16^. Alternatively, lncRNAs can interact with transcription factors or epigenetic proteins to regulate target gene transcription^14,17^.

Forkhead box O3 (FOXO3) belongs to the O subclass of the forkhead family of transcription factors and is a well-established tumor suppressor^18^. FOXO3 has been shown to control various aspects of breast cancer biology^19–21^. Song et al.^19^ reported that restoration of FOXO3 induces apoptosis and cell cycle arrest and reverses 5-fluorouracil resistance in human breast cancer cells. Sisci et al.^20^ demonstrated that overexpression of FOXO3 decreases migration, invasion, and anchorage-independent growth of estrogen receptor α-positive breast cancer cells. Multiple target genes such as CCND1 and VEGF have been proposed to mediate the anticancer activity of FOXO3^21,22^.

Although many lncRNA transcripts encoded by the 1p36 locus have been deposited in GenBank database, their biological functions have not been deciphered yet. In this study, we used small interfering RNA (siRNA) technology to deplete lncRNA candidates transcribed from 1p36 in an immortal, nontransformed breast epithelial cell line, MCF10A, with the aim to identify novel tumor-suppressing lncRNAs. The expression and function of the interested lncRNA(s) in breast cancer was explored.

## Results

### LINC01355 functions as a tumor suppressor

**Fig. 1:**
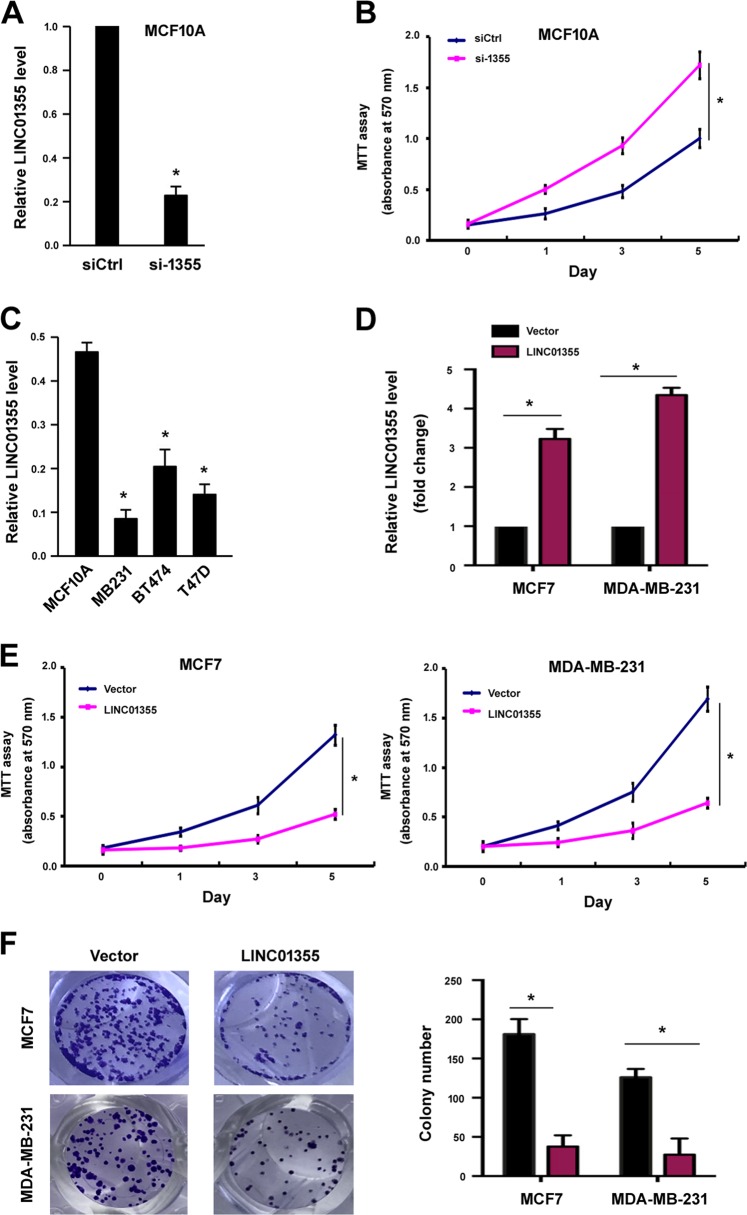
LINC01355 is downregulated and suppresses cell growth in breast cancer cells. **a** LINC01355 expression was detected by qRT-PCR in MCF10A cells transfected with control siRNA (siCtrl) or LINC01355-targeting siRNA (si-1355). **b** Cell proliferation was determined by the MTT assays at indicated time points. **c** LINC01355 expression was detected by qRT-PCR in indicated cell lines. **d** LINC01355 expression was detected by qRT-PCR in MCF7 and MDA-MB-231 cells transfected with empty vector or LINC01355-expressing plasmid. **e** Cell proliferation was determined by the MTT assays at indicated time points. **f** Colony formation assay was performed to evaluate the colony formation ability of MCF7 and MDA-MB-231 cells transfected with indicated constructs. Left, representative images of colonies stained with crystal violet. *P < 0.05

In this study, we included 37 candidate lncRNAs that are transcribed from the 1p36 locus. Among them, LINC01342, PINK1-AS, PIK3CD-AS1, LINC01772, and LINC01355 were downregulated in MCF7 cells compared with MCF10A cells (Supplementary Figure S1). Specific siRNAs were used to deplete the five lncRNAs in MCF10A cells. Of note, knockdown of LINC01355 (Fig. 1a) led to a significant increase in the proliferation of MCF10A cells (Fig. 1b). However, depletion of the other lncRNAs tested had minimal effect on the proliferation of MCF10A cells (data not shown). As breast cancer cell lines showed a general downregulation of LINC01355 (Fig. 1c), we performed LINC01355 gain-of-function studies in both MCF7 and MDA-MB-231 cells. We found that overexpression of LINC01355 (Fig. 1d) significantly inhibited the proliferation (Fig. 1e) and colony formation (Fig. 1f) of breast cancer cells. Several studies have indicated that multiple pathways including PI3K/Akt, ERK, and STAT3 are engaged in breast cancer growth^23–25^. Next, we examined the effect of LINC01355 overexpression on the activation of PI3K/Akt, ERK, and STAT3 signaling. The results showed that ectopic expression of LINC01355 did not change the activation status of PI3K/Akt, ERK, or STAT3 (Supplementary Figure S2).

### LINC01355 overexpression suppresses breast cancer cell growth in vivo

**Fig. 2:**
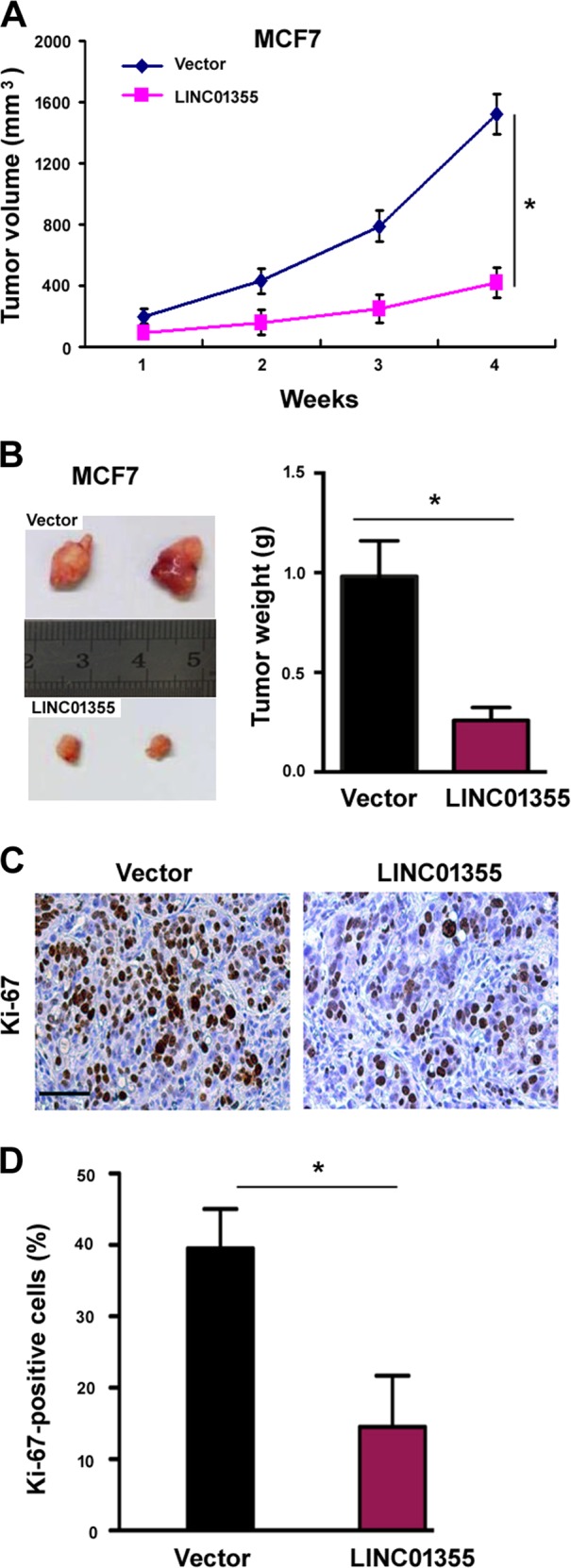
LINC01355 overexpression suppresses breast cancer cell growth in vivo. **a** MCF7 cells were stably transfected with empty vector or LINC01355-expressing plasmid and inoculated into nude mice. Growth curves were plotted based on tumor volume. **b** Left, representative images of xenograft tumors. Right, final tumor weight was determined 4 weeks after cell injection. **c** Representative images of Ki-67 immunostaining in tumor samples. Scale bar = 100 μm. **d** Quantification of Ki-67 positive cells for each group. *P < 0.05

Next, we tested whether LINC01355 can antagonize the tumorigenesis of breast cancer cells in vivo. To address this, LINC01355-overexpressing and control MCF7 cells were inoculated into female nude mice, and tumor growth was monitored. We noted that the xenograft tumor growth was significantly reduced in the LINC01355 group compared with the control group (*P* < 0.05; Fig. 2a). Also, final tumor weight was 68% lower in the LINC01355 group than that in the control group (*P* < 0.05; Fig. 2b). Consistent with this, tumor tissues from LINC01355-overexpressing cells had reduced proliferation (Fig. 2c and d), as determined by immunohistochemical analysis. Taken together, LINC01355 acts as a tumor suppressor in breast cancer.

### LINC01355 induces cell cycle arrest at the G0/G1 phase by repressing CCND1 expression

**Fig. 3:**
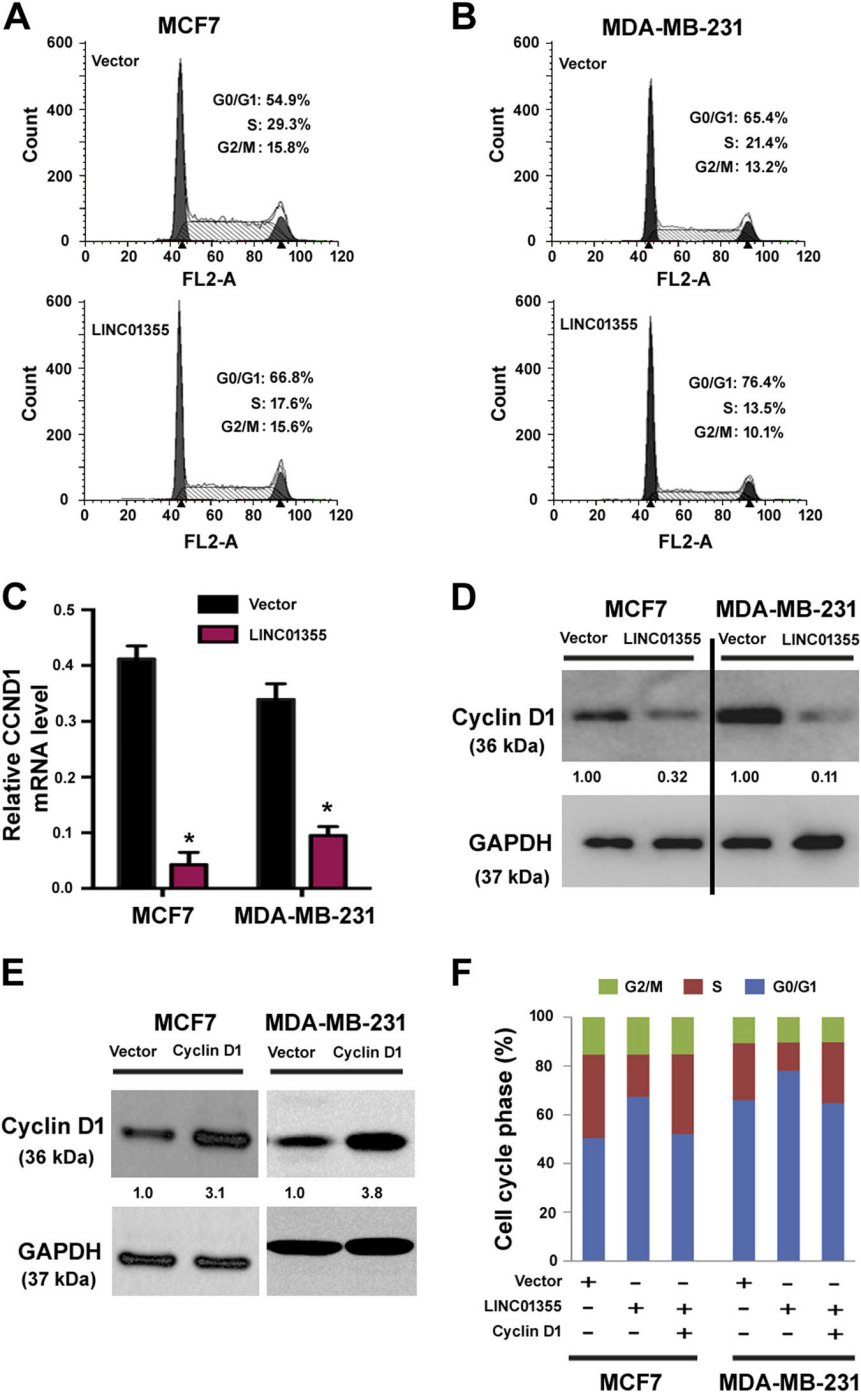
LINC01355 induces cell cycle arrest at the G0/G1 phase by repressing CCND1 expression. **a**, **b** Flow cytometric analysis of cell cycle progression in MCF7 and MDA-MB-231 cells transfected with empty vector or LINC01355-expressing plasmid after PI staining. **c** qRT-PCR analysis of CCND1 mRNA expression in MCF7 and MDA-MB-231 cells transfected with indicated constructs. *, *P* < 0.05 vs. vector-transfected cells. **d**, **e** Western blot analysis of cyclin D1 protein levels in MCF7 and MDA-MB-231 transfected with indicated constructs. Numbers represent fold change in protein levels. **f** Flow cytometric analysis of cell cycle progression in MCF7 and MDA-MB-231 cells transfected with indicated constructs

Next, we explored the effect of LINC01355 dysregulation on cell cycle progression. Of note, LINC01355-overexpressing MCF7 (Fig. 3a) and MDA-MB-231 (Fig. 3b) cells exhibited an accumulation at the G0/G1 phase and concomitant reduction at the S phase, indicating G0/G1 cell cycle arrest. Real-time PCR analysis of cell cycle related genes revealed that CCND1 expression was reduced in LINC01355-overexpressing cells relative to control cells (Fig. 3c). Consistent with the reduction of CCND1 transcripts, we also found a decline in the cyclin D1 protein level after LINC01355 overexpression (Fig. 3d). However, the other genes tested, i.e., CCND3, CCNE2, CDK4, CDK6, CDKN1A, and CDKN1B, were unaffected by LINC01355 (Supplementary Figure S3).

To validate the hypothesis that LINC01355-mediated cell cycle arrest is mainly ascribed to reduced cyclin D1 levels, we performed rescue experiments by overexpressing cyclin D1. As a result, enforced expression of cyclin D1 (Fig. 3e) significantly reversed LINC01355 overexpression-induced changes in cell cycle distribution in MCF7 and MDA-MB-231 cells (Fig. 3f). These results indicate cyclin D1 as a critical mediator for the anticancer activity of LINC01355.

### LINC01355 interacts with and stabilizes FOXO3 protein

**Fig. 4:**
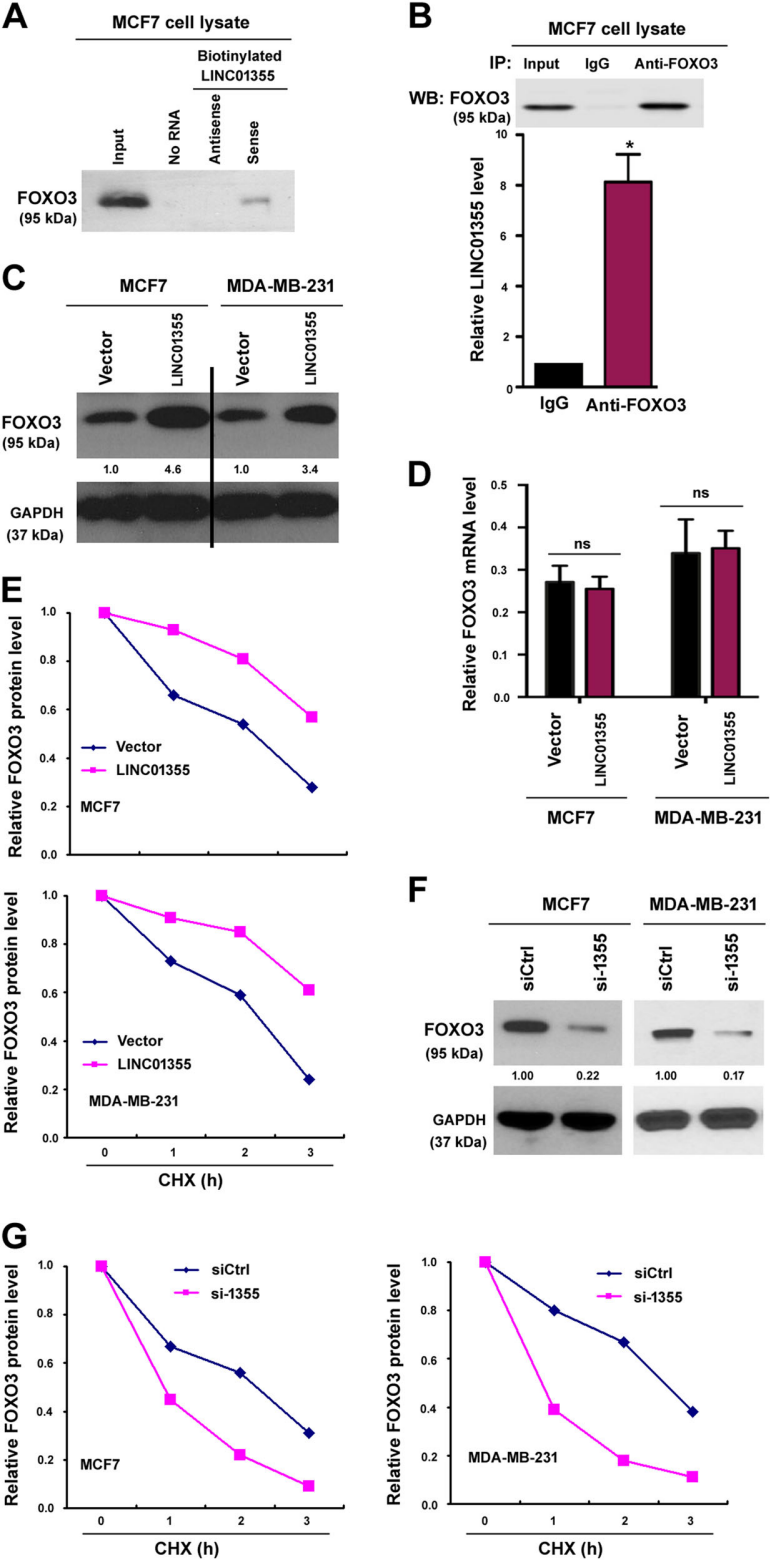
LINC01355 interacts with and stabilizes FOXO3 protein. **a** RNA pull-down assay. Western blot analysis confirmed that FOXO3 protein was pulled down by biotinylated LINC01355 sense transcript but not by antisense transcript. **b** RIP assay revealed that LINC01355 was detected in FOXO3 immunoprecipitates from MCF7 cells. Upper, western blot analysis of FOXO3 immunoprecipitates. *, *P* < 0.05 vs. control IgG immunoprecipitates. **c** Western blot analysis of FOXO3 protein levels in MCF7 and MDA-MB-231 transfected with indicated constructs. Numbers represent fold change in protein levels. **d** qRT-PCR analysis of FOXO3 mRNA expression in MCF7 and MDA-MB-231 cells transfected with indicated constructs. ns = no significance. **e** Western blot analysis of FOXO3 protein levels at different time points in transfected cells after treatment with 50 μm cycloheximide. **f** Western blot analysis of FOXO3 protein levels in MCF7 and MDA-MB-231 transfected with indicated constructs. Numbers represent fold change in protein levels. **g** Western blot analysis of FOXO3 protein levels at different time points in transfected cells after treatment with 50 μm cycloheximide

To uncover the detailed mechanism underlying the action of LINC01355, we conducted RNA pull-down assays in MCF7 cell extracts followed by proteomic analysis of LINC01355-associated proteins. The results showed that a number of proteins were highly enriched in the LINC01355 pull-down complexes. Among them, FOXO3 attracted more attention because of its broad-spectrum antitumor activity^19,20^. Western blot analysis confirmed the presence of FOXO3 in the complex pulled down by biotinylated LINC01355 sense transcript but not by antisense transcript (Fig. 4a). To validate the association between LINC01355 and FOXO3, radioimmunoprecipitation (RIP) assay was further performed. We found that LINC01355 could be detected in FOXO3 immunoprecipitates from MCF7 cells (Fig. 4b).

Next, we examined the effect of LINC01355 on the expression of FOXO3 in breast cancer cells. Notably, overexpression of LINC01355 caused an elevation in the protein level of FOXO3 in both MCF7 and MDA-MB-231 cells (Fig. 4c). However, the mRNA level of FOXO3 remained unchanged (Fig. 4d). We speculated that LINC01355-mediated upregulation of FOXO3 protein may be a consequence of enhanced protein stability. In support of this hypothesis, the half-life of FOXO3 protein was longer in LINC01355-overexpressing MCF7 cells than that in control cells (Fig. 4e). In contrast, knockdown of LINC01355 diminished the level of FOXO3 protein (Fig. 4f) and accelerated FOXO3 protein turnover (Fig. 4g). These data indicate that LINC01355 acts as a stabilizer of FOXO3 protein.

### LINC01355 promotes FOXO3-mediated transcriptional repression of CCND1

**Fig. 5:**
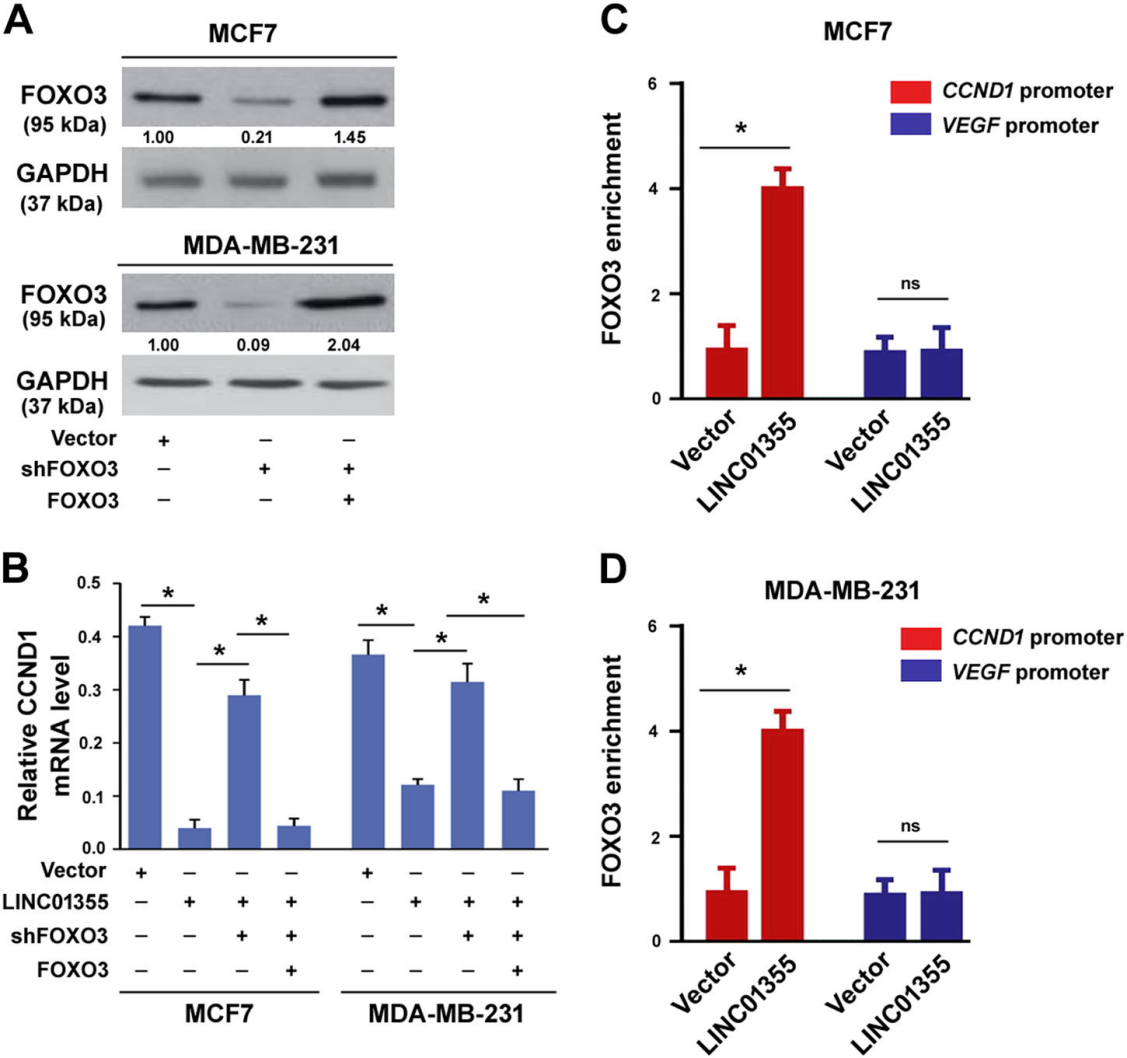
LINC01355 promotes FOXO3-mediated transcriptional repression of CCND1. **a** Western blot analysis of FOXO3 protein levels in MCF7 and MDA-MB-231 transfected with indicated constructs. Numbers represent fold change in protein levels. **b** qRT-PCR analysis of CCND1 mRNA expression in MCF7 and MDA-MB-231 cells transfected with indicated constructs. *, *P* < 0.05. **c**, **d** ChIP assays were performed in MCF7 and MDA-MB-231 transfected with indicated constructs to detect FOXO3 enrichment at the *CCND1* promoter. The *VEGF* promoter was used as a control. *P < 0.05; ns, no significance

Given that FOXO3 is a transcriptional repressor of CCND1^21^, we next checked whether FOXO3 is involved in LINC01355-dependent reduction of CCND1 in breast cancer cells. To this end, we performed FOXO3 knockdown experiments (Fig. 5a). We found that LINC01355-induced downregulation of CCND1 was remarkably impaired when FOXO3 was depleted (Fig. 5b). Moreover, re-expression of FOXO3 (Fig. 5a) restored LINC01355-mediated inhibition of CCND1 expression (Fig. 5b). Chromatin immunoprecipitation (ChIP) assay further demonstrated that LINC01355 overexpression led to a selective enrichment of FOXO3 protein at the promoter of *CCND1* (Fig. 5c and d). As a control, the FOXO3 binding to the *VEGF* promoter was not altered by LINC01355 overexpression (Fig. 5c and d). Taken together, we propose that LINC01355-induced downregulation of CCND1 depends on FOXO3 activity.

### Increased FOXO3 level is responsible for LINC01355-induced tumor suppression

**Fig. 6:**
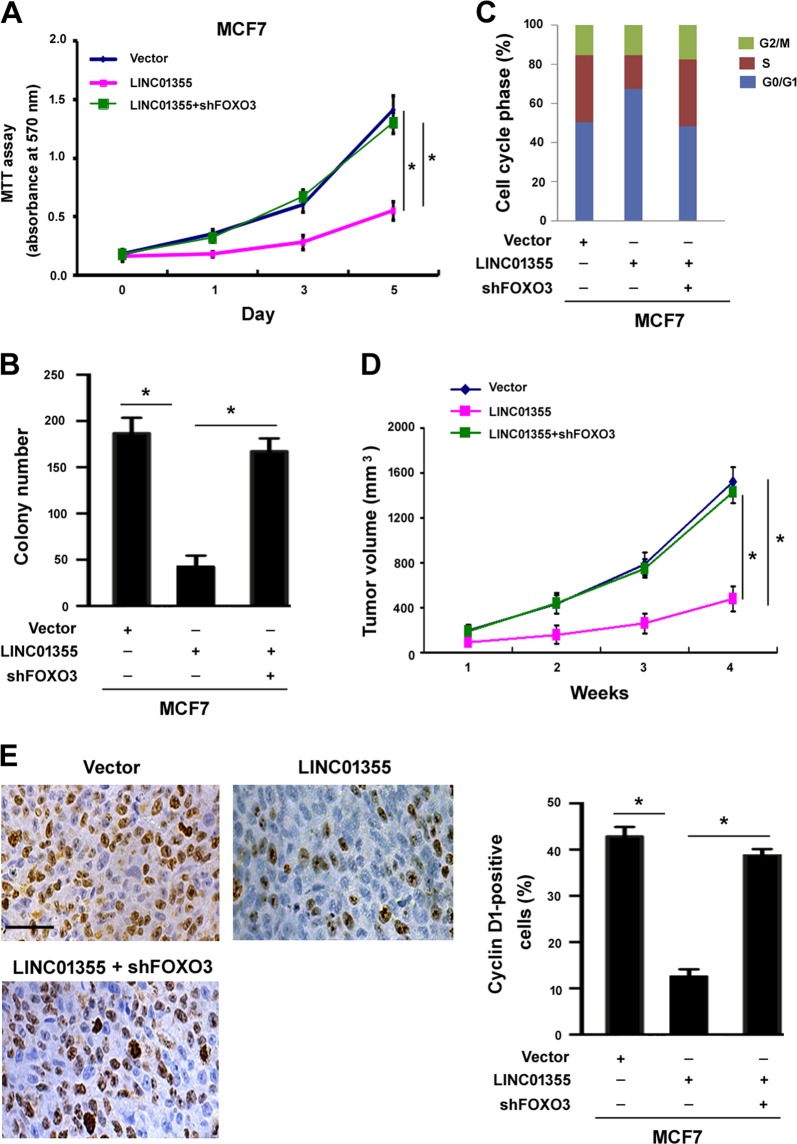
Increased FOXO3 level is responsible for LINC01355-induced tumor suppression. **a** MCF7 cells transfected with indicated constructs were tested for proliferation at different time points by the MTT assays. **b** Colony formation assays were performed in MCF7 cells transfected with indicated constructs. **c** Flow cytometric analysis of cell cycle progression in MCF7 cells transfected with indicated constructs. **d** MCF7 cells were stably transfected with indicated constructs and inoculated into nude mice. Growth curves were plotted based on tumor volume. **e** Representative images of cyclin D1 immunostaining in tumor samples. Scale bar = 60 μm. **d** Quantification of cyclin D1 positive cells for each group. *P < 0.05

We next investigated the role of FOXO3 in the growth suppression induced by LINC01355. As shown in Fig. 6a and b, LINC01355-mediated inhibition of breast cancer cell proliferation and clonogenicity were reversed by knockdown of FOXO3. Analysis of cell cycle distribution showed that depletion of FOXO3 allowed LINC01355-overexpressing MCF7 cells to re-enter into cell cycle (Fig. 6c). In vivo studies confirmed that FOXO3 deficiency abolished the suppression of MCF7 xenograft tumor growth induced by LINC01355 (Fig. 6d). Immunohistochemical analysis of cyclin D1 demonstrated that depletion of FOXO3 relieved the inhibition of cyclin D1 caused by LINC01355 (Fig. 6e). These observations suggest that FOXO3 is required for LINC01355-induced anticancer activity.

### LINC01355 expression is correlated with clinical features and CCND1 mRNA levels in breast cancer

**Fig. 7:**
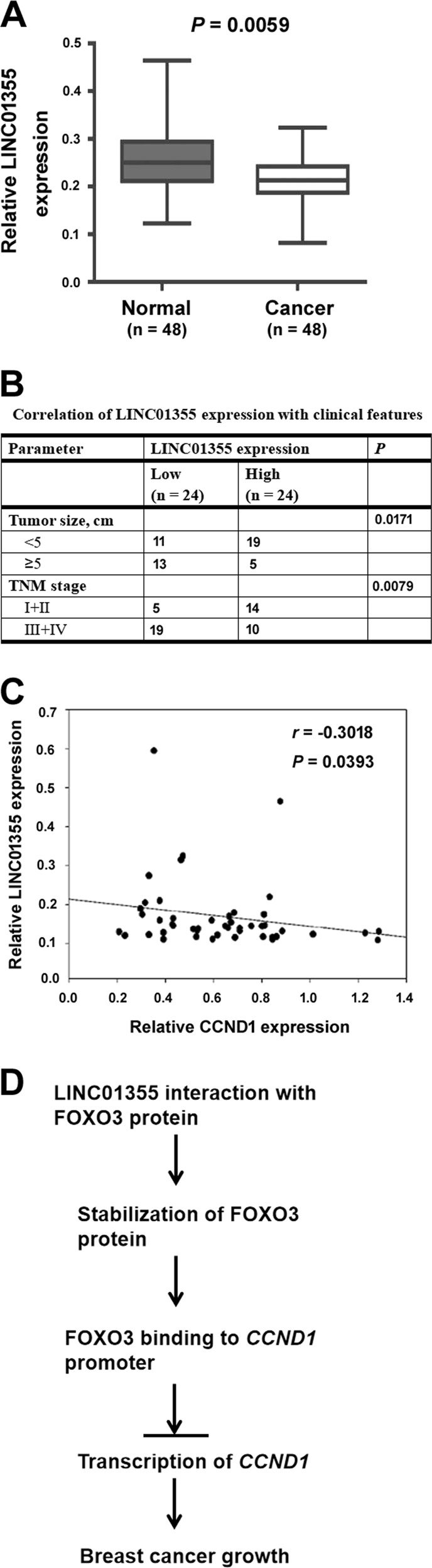
LINC01355 expression is correlated with clinical features and CCND1 mRNA levels in breast cancer. **a** qRT-PCR analysis of LINC01355 expression in paired breast cancer tissues and normal breast tissues. **b** Downregulation of LINC01355 was significantly correlated with larger tumor size and advanced clinical stage of breast cancer. **c** A negative correlation was detected between LINC01355 and CCND1 expression in breast cancer tissues. **d** Schematic illustration for the mechanisms by which LINC01355 interacts with FOXO3 to suppress breast cancer growth

Next, we evaluated the expression and clinical significance of LINC01355 in breast cancer. We found that LINC01355 was significantly downregulated in breast cancer tissues relative to normal breast tissues (*P* = 0.0059; Fig. 7a). Downregulation of LINC01355 was significantly correlated with larger tumor size (*P* = 0.0171) and advanced clinical stage (*P* = 0.0079) of breast cancer (Fig. 7b). However, the expression status of LINC01355 was not associated with age, lymph node metastasis, ER, or PR status (data not shown). The Spearman correlation analysis demonstrated a negative correlation between LINC01355 and CCND1 expression in breast cancer tissues (*r* = −0.3018, *P* = 0.0393; Fig. 7c).

## Discussion

The 1p36 locus is frequently deleted in breast cancer^7^, suggesting its involvement in cancer development and progression. Previously, an array of protein-coding tumor suppressor genes localized at the region have been identified^5,6^. For example, CHD5 is downregulated and shows growth-suppressive activity in breast cancer^26^. Similarly, PER3 and RIZ1 exert inhibitory effects on aggressive phenotypes of breast cancer cells^5,27^. In this work, we focus on lncRNAs embedded in the 1p36 locus. We demonstrate that LINC01355 expression is underexpressed in breast cancer tissues and cell lines compared with their normal equivalents. Clinically, LINC01355 downregulation is significantly associated with tumor size and TNM stage in breast cancer. Functionally, LINC01355 knockdown renders a proliferation advantage to breast cancer cells, whereas ectopic expression of LINC01355 suppresses the proliferation and colony formation of breast cancer cells. In vivo data further validate the suppressive activity of LINC01355 in breast cancer tumorigenesis. LINC01355 overexpression leads to reduced proliferation and increased apoptosis in MCF7 xenograft tumors. Taken together, we provide first evidence that LINC01355 acts as a tumor suppressor in breast cancer.

Our data further demonstrate that LINC01355 overexpression causes a cell cycle arrest at the G0/G1 phase. Biochemically, LINC01355 overexpression inhibits the expression of cyclin D1 in breast cancer cells. Cyclin D1 is regarded as a key regulator of G1/S cell cycle transition through via the ability to bind and activate CDK4 and CDK6 kinases^28^. We found that overexpression of cyclin D1 reverses the accumulation of cells at the G0/G1 phase caused by LINC01355. In agreement with the in vitro findings, there is an inverse correlation between LINC01355 and CCND1 expression. These results suggest that downregulation of cyclin D1 is an important mechanism responsible for the growth-suppressive activity of LINC01355.

Several mechanisms have been proposed to be involved in the regulation of cyclin D1^29,30^. For example, the deubiquitylase ubiquitin-specific peptidase 22 post-translationally regulates cyclin D1 expression by preventing proteasome-mediated degradation^29^. LIN28A has been shown to promote the expression of cyclin D1 by inhibiting the production of let-7^30^. A number of transcription factors such as FOXO3 participate in the transcriptional regulation of CCND1^21^. Here, we provide evidence for the association between LINC01355 and FOXO3 protein in breast cancer cells. LINC01355 overexpression increases the level of FOXO3 protein but not mRNA, implying the post-transcriptional regulation of FOXO3. Indeed, the interaction with LINC01355 enhances FOXO3 protein stability, as evidenced by prolonged half-life of FOXO3 protein. Besides increased protein stability, LINC01355 selectively enhances the binding of FOXO3 protein to the promoter of *CCND1*. As a negative control, LINC01355 fails to impact the FOXO3 binding to *VEGF* promoter. These data collectively point toward that LINC01355 acts as a stabilizer of FOXO3 and downregulates CCND1 through enhancement of FOXO3-mediated transrepression. Consistent with the finding that knockdown of FOXO3 abrogates LINC01355-induced downregulation of CCND1, FOXO3 depletion reverses the inhibitory effect of LINC01355 on breast cancer cell proliferation and tumorigenesis. Therefore, we propose that the FOXO3/CCND1 axis plays an essential role in LINC01355-mediated tumor suppression (Fig. 7d).

It has been previously reported that the USP9x deubiquitinase is involved in the stabilization of FOXO3 protein^31^. We speculate that LINC01355 binding likely promotes the association between FOXO3 and deubiquitinases, thus reducing proteasomal degradation of FOXO3. Ongoing studies are conducted to identify the key mediator for LINC01355-mediated stabilization of FOXO3. In addition, it remains to be determined whether LINC01355 can exert its tumor-suppressing activity in other cancers besides breast cancer.

In conclusion, our study identifies LINC01355 as a novel tumor suppressor. Downregulation of LINC01355 contributes to aggressive phenotype of breast cancer. Re-expression of LINC01355 suppresses the growth and tumorigenesis of breast cancer cells, which involves the stabilization of FOXO3 and enhancement of FOXO3-mediated transrepression of CCND1. Therefore, strategies to induce LINC01355 expression may have therapeutic potential in breast cancer.

## Materials and methods

### Cell lines

All cell lines were obtained from the Type Culture Collection of the Chinese Academy of Sciences (Shanghai, China). The cells were cultured in RPMI-1640 medium (Lonza, Verviers, Belgium) supplemented with 10% fetal bovine serum (Sigma-Aldrich, St. Louis, MO, USA), penicillin (100 U/ml), and streptomycin (100 μg/ml). Cell lines were validated to be free of mycoplasma.

### Quantitative real-time PCR (qRT-PCR) analysis

Total RNA was isolated from cell lines and tissue samples using Trizol (Invitrogen, Carlsbad, CA, USA) following manufacturer's instructions. For qRT-PCR, RNA was reverse transcribed using the High-Capacity CDNA Reverse Transcription Kit (Invitrogen). PCR was performed using the Power SYBR Master Mix (Applied Biosystems, Foster City, California, USA). The primers used are listed in Table 1. Relative expression of each target gene was normalized to GAPDH mRNA level and calculated by the 2^−ΔΔCt^ method^32^.

**Table 1:** List of oligonucleotides used in the study

qRT-PCR primers	Sequence
LINC01342	Forward: GTTTGACTTGTTCAGGCACA Reverse: GTCCTCCAAAGACGAGAACAG
PINK1-AS	Forward: GAGCCTGTTGGCCAACAAAGT Reverse: GTTGAGACTGTGTTAACAGATG
PIK3CD-AS1	Forward: AGCAGTAAACCTTCCCCTCC Reverse: TCTTGAACCCCACCAGACTC
LINC01772	Forward: CTTACTTGAGGTTAGGAGTTC Reverse: GTACAGTGGCATGATCTCAG
LINC01355	Forward: CTGCTCTAGCCCCTAAAGATAG Reverse: GGATTCCAAATGACACATTCCT
CCND1	Forward: GCTGCGAAGTGGAAACCATC Reverse: CCTCCTTCTGCACACATTTGAA
FOXO3	Forward: TCTACGAGTGGATGGTGCGTT Reverse: CGACTATGCAGTGACAGGTTGTG
GAPDH	Forward: ACGGATTTGGTCGTATTGGGC Reverse: TTGACGGTGCCATGGAATTTG
ChIP qPCR primers	
CCND1	Forward: GAAATCCCTTTAACTTTTAG Reverse: GAATCTCAGCGACTGCATCT
VEGF	Forward: TCCGGGTTTTATCCCTCTTC Reverse: TCTGCTGGTTTCCAAAATCC
shRNA	
shFOXO3	CCGGCATGTTCAATGGGAGCTTGGACTCGAGTCCAAGCTCCCATTGAACATG TTTTT
siRNA	
siCtrl	AGCUUCAUAAGGCGCAUAG
si-1355 #1	CCUCAACUCCUGCUUCCAA
si-1355 #2	CCAAUUGCAUAAUAUGGUU
si-1355 #3	CUCACACUGAAUUGAUCAA

*siCtrl* control siRNA, *si-1355* LINC01355-targeing siRNA

### Gene knockdown by RNA interfering technology

For gene knockdown experiments, gene-specific siRNA or short hairpin RNA were synthesized by Hanyu Biotech (Beijing, China). Target sequences are listed in Table 1. Cells were transfected using Lipofectamine 3000 (Invitrogen) following the manufacturer’s instructions. Twenty-four hours after transfection, cells were subjected to further experiments. FOXO3 stably knockdown MCF7 cells were generated after puromycin selection. Knockdown efficiency was determined using qRT-PCR or western blot analyses.

### Plasmid construction and transfection

The plasmids encoding LINC01355 (NR_110616), CCND1 (NM_053056), and FOXO3 (NM_001455) were purchased from Hanyu Biotech. The inserts were confirmed by sequencing. The plasmids were transfected into cells using Lipofectamine 3000. For generation of stable cell lines, transfected cells were selected with 800 μg/ml G418 (Sigma-Aldrich).

### Western blot analysis

Cells were lysed in ice-cold buffer (50 mM Tris-HCl, pH 7.4, 150 mM NaCl, 1 mM EDTA, 1 mM EGTA, 1% NP-40, 0.1% sodium dodecyl sulfate (SDS), 1% triton X-100) containing protease/phosphotase inhibitor cocktails (Sigma-Aldrich). Protein concentration was quantified using the BCA protein assay (Bio-Rad Laboratories, Hercules, CA, USA). Equal amounts of protein were separated by SDS-PAGE and transferred to nitrocellulose membranes. The membranes were incubated with 5% fat-free milk at room temperature for 1 h to block nonspecific binding, and probed with the primary antibodies recognizing cyclin D1, FOXO3, and GAPDH (Cell Signaling Technology, Inc., Beverly, MA, USA). After washing, the blots were incubated with peroxidase-labeled secondary antibodies (Cell Signaling Technology) and developed using an enhanced chemiluminescence detection kit (Amersham Biosciences, Inc., Piscataway, NJ, USA). The intensities of the bands were quantified by densitometry using Quantity One software (Bio-Rad Laboratories).

### Cell proliferation assay

Cells were seeded onto 96-well plates (5 × 10^3^ cells/well) and cultured for 1, 3, and 5 days. The 3-[4,5-dimethylthiazol-2-yl]-2,5 diphenyl tetrazolium bromide (MTT) solution (Sigma-Aldrich) was added to each well and incubated for 4 h at 37 °C in a humidified atmosphere. The MTT formazan precipitate was dissolved in dimethyl sulfoxide. Absorbance was measured at 570 nm. Cell growth curves were plotted.

### Colony formation assay

Colony formation assay was performed as described previously^33^. In brief, cells were plated in six-well plates (600 cell/well) and allowed to grow for an additional 12 days. The colonies were fixed in 70% ethanol and then stained with 0.5% crystal violet staining solution. The colonies were counted under a microscope.

### In vivo studies

Female Balb/c nude mice (5-week-old) were used for xenograft experiments. MCF7 cells stably transfected with indicated constructs were subcutaneously inoculated into nude mice (3 × 10^6^ cells/mouse). Tumor volume was determined every week for 4 weeks after cell injection. The animals were then sacrificed, and xenograft tumors were weighed. Xenograft tumor samples were formalin-fixed, paraffin-embedded, and sectioned. Tissue sections were deparaffinized, rehydrated, and incubated with anti-Ki-67 and anti-cyclin D1 antibodies. Diaminobenzidine was used for colorimetric development.

### Flow cytometric analysis of cell cycle distribution

Cells were fixed in ice-cold 70% ethanol and treated with 50 µg/mL propidium iodide (Sigma-Aldrich) for 30 min in the dark. The cell cycle profile was assayed using a flow cytometer (BD Biosciences, San Jose, CA, USA).

### RNA pull-down assay

RNA pull-down were performed as described previously^34^. In brief, biotin-labeled LINC01355 (sense or antisense) was generated using the Biotin RNA Labeling Mix containing T7 RNA polymerase (Roche Diagnostics, Indianapolis, IN, USA) and incubated with MCF7 cellular lysates. Streptavidin agarose beads were added to retrieve protein immunoprecipitates. LINC01355-immunoprecipitated proteins were subjected to mass spectrometric analysis and western blot analysis.

### RNA immunoprecipitation (RIP) assay

RIP experiments were performed as described previously^35^. In brief, MCF7 cells were lysed, and the lysates were immunoprecipitated with anti-FOXO3 antibody or control isotype IgG, followed by RNA recovery. The co-precipitated RNAs were detected by qRT-PCR.

### ChIP assay

ChIP assay was performed as described previously^36^. In brief, cells transfected with empty vector or LINC01355-expressing plasmids were cross-linked using 1% formaldehyde. Chromatin was isolated and sonicated to small fragments. Anti-FOXO3 antibodies were used to precipitate DNA-protein complexes. ChIP DNA was analyzed by qRT-PCR. The primers are listed in Table 1.

### Determination of FOXO3 protein turnover

Analysis of protein turnover was performed as described previously^37^. In brief, cells were transfected with empty vector or LINC01355-expressing plasmids and then treated with 50 μm cycloheximide (Sigma-Aldrich) to inhibit protein synthesis. Cells were lysed at indicated time points and subjected to western blot analysis.

### Patients and samples

Forty-eight breast cancer tissue samples and adjacent noncancerous breast tissues were collected from breast cancer patients who underwent surgical resection between 2016 and 2017 at our hospital. Diagnosis was confirmed by histology. No patient received pre-operative anticancer treatments. Tissue samples were snap-frozen in liquid nitrogen and stored at −80 °C prior to RNA isolation.

### Statistical analysis

Differences among groups were analyzed by the Student's *t* test, one-way analysis of variance, or Mann–Whitney *U* test. The Spearman correlation analysis was used to analyze the relationship between LINC01355 and CCND1 expression in breast cancer tissues. The Chi-square test was used to analyze the associations of LINC01355 expression with pathologic features of breast cancer. *P* < 0.05 was considered significant.

## Supplementary information

Supplementary Data.
