# Supplementary material for: LINC01355 suppresses breast cancer growth through FOXO3-mediated transcriptional repression of CCND1
Source: Cell Death Dis. 2019 Jun 26;10(7):502. doi: 10.1038/s41419-019-1741-8 (PMC6594972; doi:10.1038/s41419-019-1741-8)
Supplement: Supplementary file 1 — Supplementary Data. [file 41419_2019_1741_MOESM1_ESM.doc]

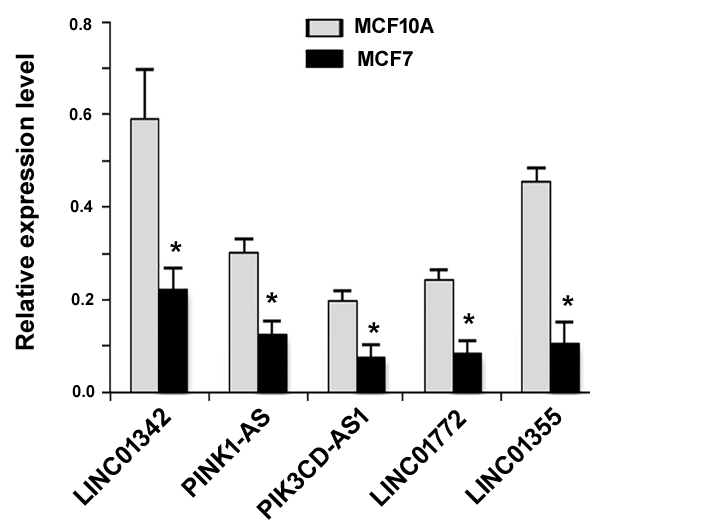


**Supplementary Figure S1.** Measurement of the expression of lncRNAs in MCF10A and MCF7 cells by quantitative real-time PCR analysis. ^*^*P* < 0.05 vs. MCF10A cells.


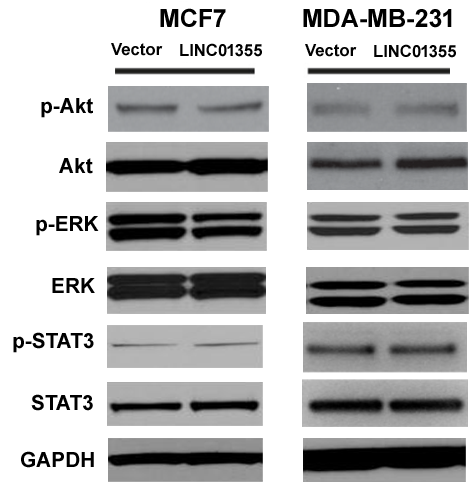


**Supplementary Figure S2.** Western blot analysis of indicated proteins in both MCF7 and MDA-MB-231 cells transfected with empty vector or LINC01355-expressing plasmid.


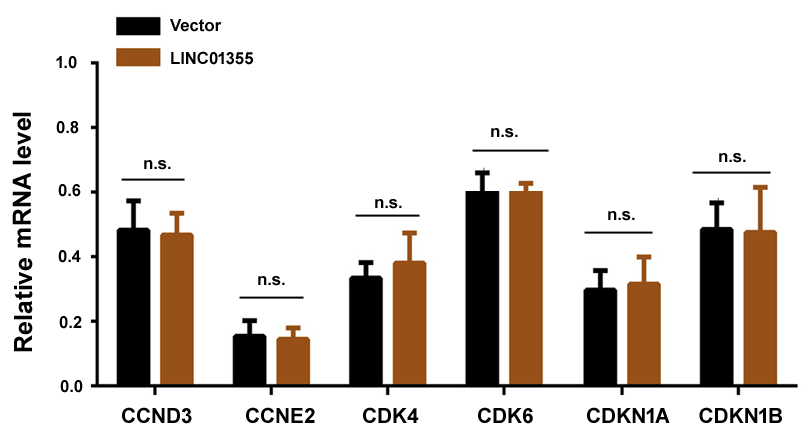


**Supplementary Figure S3.** Real-time PCR analysis of indicated genes in MCF7 cells transfected with empty vector or LINC01355-expressing plasmid. n.s. indicates no significance.
